# Life as Counterfactual Geometry: An Adversarial Theory of Biological Function

**DOI:** 10.3390/e28030255

**Published:** 2026-02-26

**Authors:** Călin Gheorghe Buzea, Florin Nedeff, Diana Mirilă, Valentin Nedeff, Maricel Agop, Lăcrămioara Ochiuz, Lucian Dobreci, Decebal Vasincu

**Affiliations:** 1 National Institute of Research and Development for Technical Physics, IFT Iași, 700050 Iași, Romania; calinb2003@yahoo.com; 2 Clinical Emergency Hospital “Prof. Dr. Nicolae Oblu” Iași, 700309 Iași, Romania; 3 Department of Environmental Engineering, Mechanical Engineering and Agritourism, Faculty of Engineering, “Vasile Alecsandri” University of Bacău, 600115 Bacău, Romania; florin_nedeff@ub.ro (F.N.); vnedeff@ub.ro (V.N.); m.agop@yahoo.com (M.A.); dobreci.lucian@ub.ro (L.D.); 4 Faculty of Medicine, “Grigore T. Popa” University of Medicine and Pharmacy Iași, 700115 Iași, Romania; lacramioara.ochiuz@umfiasi.ro (L.O.); decebal.vasincu@umfiasi.ro (D.V.)

**Keywords:** counterfactual geometry, adversarial stabilization, information geometry, adaptive dynamics, disease instability, systems medicine

## Abstract

Living systems exhibit anticipation, adaptability, and resilience that cannot be fully explained by stimulus–response models, static homeostasis, or convergence-based optimization. This work addresses this gap by proposing a theoretical framework in which a central aspect of biological function is understood through the geometry and stability of distributions over unrealized but accessible future trajectories. We formalize these distributions as a *counterfactual manifold*, defined as a probabilistically supported subset of path space induced by a system’s effective internal dynamics. Using tools from information geometry and dynamical systems theory, we analyze adaptive systems that modify the laws governing their own future trajectories and construct explicit dual-channel adversarial dynamics that couple processes expanding future possibilities with antagonistic processes enforcing feasibility constraints. We show that adaptive systems of this kind are generically unstable, tending toward either collapse of accessible futures or unbounded sensitivity to perturbation. Constructive adversarial dynamics are sufficient to stabilize counterfactual geometry without requiring convergence to a fixed point. A minimal adversarial model reveals three generic regimes: collapse, runaway sensitivity, and bounded non-convergent regulation. The framework yields operational, falsifiable predictions through measurable proxies based on response diversity, perturbation sensitivity, recovery geometry, and boundary residence, allowing these regimes to be discriminated using finite observations without reconstructing underlying state-space dynamics. Interpreting disease as instability of counterfactual geometry provides a unifying language for understanding rigidity, volatility, and context dependence across biological domains. Rather than replacing mechanistic models, the proposed framework offers a higher-level geometric and dynamical perspective in which such models can be embedded and compared, shifting attention from component-level dysfunction to the stability of biological futures and establishing a principled foundation for analyzing disease, intervention, and adaptability across scales.

## 1. Introduction

Living systems exhibit a capacity for anticipation, adaptability, and resilience that fundamentally distinguishes them from passive physical matter. Organisms routinely act in ways that are better explained by reference to *possible futures* than to immediate stimuli: immune systems prepare for pathogens not yet encountered, brains plan actions in hypothetical environments, and tumors evolve resistance strategies before therapeutic pressure is fully realized [1,2]. Despite this, dominant frameworks in biology and medicine continue to model function largely in terms of stimulus–response mappings, feedback regulation, or component-level optimization, offering limited insight into how living systems manage uncertainty, novelty, and long-term survival.

Classical homeostasis, originating in the work of Cannon [3], conceptualizes biological stability as the maintenance of internal variables around fixed set points. While later developments such as allostasis [4] and predictive processing [5,6,7] have extended this view to include adaptive regulation and expectation-based control, these approaches remain fundamentally trajectory-centric: biological behavior is described as movement along a single realized path, modulated by feedback or prediction error minimization. Such descriptions struggle to account for phenomena in which *the structure of unrealized possibilities*—rather than the realized state alone—appears to determine function and dysfunction.

In neuroscience, predictive coding and the free energy principle have provided influential accounts of perception and action as inference under uncertainty, emphasizing internal generative models and error correction [5,6,7]. However, these theories primarily address how systems infer the causes of current sensory data, not how they maintain a *diverse yet constrained repertoire of future trajectories*. Empirically, healthy neural systems are characterized by rich variability, metastability, and bounded fluctuations [8,9], whereas pathological states often manifest as either excessive rigidity (e.g., stereotyped behavior, depressive states, neurodegeneration) or uncontrolled variability (e.g., hallucinations, seizures, manic states). These observations suggest that biological function may depend less on convergence to optimal states than on maintaining structured access to multiple potential futures.

Related insights have emerged in dynamical systems biology, where attractor landscapes have been used to describe cell differentiation, immune responses, and disease progression [10,11]. While these models capture multistability and state transitions, they typically represent biological possibilities as static features of a fixed landscape. In contrast, living systems actively *reshape* their own landscapes through learning, adaptation, and interaction with the environment. The space of possible futures is therefore neither static nor externally imposed, but internally generated and continuously updated.

In parallel, advances in artificial intelligence—particularly generative models—have highlighted the computational importance of explicitly representing and sampling from distributions over possible outcomes. Generative adversarial networks and related architectures demonstrate that rich generative capacity alone is insufficient for stable learning: without constraint, generative systems diverge or collapse, whereas overly strong constraints suppress exploration and adaptability [12]. Although these methods were developed for engineering purposes, they expose a deeper computational principle: stable representation of complex possibility spaces requires internal opposition between generative and constraining processes.

Strikingly, this principle mirrors longstanding but fragmented observations in biology. Hemispheric specialization and competition in the brain [8], regulatory opposition within immune signaling networks, and ecological constraints on tumor evolution all suggest that internal conflict, rather than harmony, is a prerequisite for stability. However, no general theoretical framework currently exists that treats this opposition as a *foundational organizing principle* of living systems.

**Figure 1:**
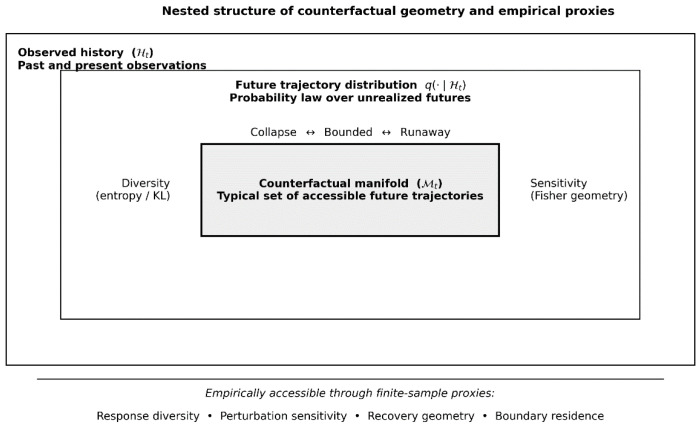
Nested structure of counterfactual geometry and empirical proxies. Observed histories $H_{t}$ induce a probability distribution $q\left(\cdot |H_{t}\right)$ over unrealized future trajectories. The counterfactual manifold $M_{t}$ is defined as the typical set of accessible futures under this distribution and is characterized by its diversity (e.g., entropy or Kullback–Leibler divergence) and sensitivity (e.g., Fisher information geometry). Regimes such as collapse, bounded regulation, and runaway sensitivity correspond to qualitative changes in the geometry and stability of $M_{t}$. Although $M_{t}$ is not directly observable, its properties can be assessed through finite-sample empirical proxies, including response diversity, perturbation sensitivity, recovery geometry, and boundary residence.

Here we propose such a framework. We introduce the concept of a counterfactual manifold: a structured, probabilistically supported space of unrealized future trajectories internally maintained by a biological system. Figure 1 provides a schematic overview of the object hierarchy underlying the framework, from observed histories to distributions over unrealized futures and the induced counterfactual manifold. It also clarifies the distinction between the geometric objects defined by the theory and the finite-sample empirical proxies through which their stability and regimes can be assessed. We argue that an essential and previously underappreciated aspect of biological function lies in preserving the geometry and stability of this manifold under continual perturbation, rather than solely in optimizing any single state variable.

This claim is intended as a high-level descriptive principle applicable to a broad class of adaptive systems, not as a universal or exclusive definition of biological function. Using tools from information geometry and dynamical systems theory, we show that maintaining a viable counterfactual manifold requires adversarial coupling between internal processes that expand the space of possibilities and processes that constrain those possibilities according to physical, energetic, and historical limits.

Within this framework, health corresponds to bounded, non-convergent dynamics on the counterfactual manifold, characterized by sufficient diversity of futures, realistic constraint satisfaction, and controlled sensitivity to perturbation [8,9,13]. Disease, by contrast, emerges as a small number of geometric failure modes: collapse of accessible futures, uncontrolled expansion, fragmentation into isolated modes, or excessive curvature leading to fragility. Importantly, these failure modes cut across traditional diagnostic boundaries, offering a unified language for understanding disorders of cognition, immunity, growth, and degeneration.

This perspective shifts the focus of medicine from restoring normal values or suppressing abnormal components to stabilizing the geometry of biological futures. Interventions—pharmacological, surgical, radiative, or behavioral—are reinterpreted as operators that reshape the counterfactual manifold or alter the balance between generative and constraining dynamics. From this viewpoint, the same intervention may be therapeutic or harmful depending on the pre-intervention geometry of the system, providing a principled explanation for context-dependent treatment effects and variability in patient response.

The aim of this work is not to replace existing mechanistic or clinical models, but to provide a higher-level theoretical substrate in which they can be coherently embedded. By introducing explicit geometric and dynamical quantities—such as counterfactual entropy, manifold curvature, and adversarial imbalance—we establish a foundation for measurable, falsifiable, and intervention-relevant descriptions of biological function. In doing so, we seek to move beyond static notions of regulation and toward a physics of living systems grounded in the structure and stability of unrealized futures.

Scope, novelty, and testability. The framework introduced here is formally distinct from inference-based or control-theoretic accounts that optimize scalar objectives over present or past states. Because such objectives admit Lyapunov functions, they generically imply convergence or fixation. As a consequence, any such formulation generically drives the conditional future law itself toward a fixed point (or effectively fixed manifold), making sustained bounded, non-convergent regulation of future-distribution diversity and sensitivity structurally impossible without external forcing, explicit time dependence, or fine-tuned degeneracy.

By contrast, the present theory operates directly on the geometry of future-conditioned path distributions and shows that stabilizing this geometry is not expressible as optimization of any single scalar functional, but instead requires irreducibly adversarial (saddle-point) dynamics with non-gradient components. Importantly, the framework is falsifiable: it predicts that stable biological function is necessarily associated with bounded diversity and bounded sensitivity of future responses, yielding observable signatures in perturbation–response statistics, variability, and recovery geometry. The theory is intended as a high-level descriptive and explanatory framework, not as a universal or exclusive account of biological function.

Outside scope. The framework developed here applies specifically to adaptive systems that (i) maintain a conditional distribution over multiple accessible future trajectories, (ii) modify the parameters governing that distribution through internal dynamics, and (iii) do so under finite resource or capacity constraints. Systems that realize only a single admissible future (purely reactive dynamics), systems whose future-conditioned law is externally fixed and not endogenously modified, or systems whose adaptation depends solely on realized trajectories without sensitivity to unrealized alternatives fall outside the explanatory scope of counterfactual stabilization as defined in this work.

Structure of the paper. Section 2 formalizes counterfactual manifolds as probabilistic objects on path space and introduces geometric quantities—relative entropy and Fisher information—that characterize diversity and sensitivity of future distributions. Section 3 shows why adaptive systems that modify their own future-generating dynamics are generically unstable and introduces adversarial stabilization as a sufficient mechanism for maintaining bounded counterfactual geometry, culminating in a minimal constructive model, explicit regime taxonomy, and operational proxies. Section 4 reframes disease as instability of counterfactual geometry and identifies generic failure modes that cut across traditional diagnostic categories. Section 5 develops domain instantiations of the framework across biological systems, including neural, immune, oncological, and developmental contexts. Section 6 interprets medical interventions as geometric operators acting on future distributions and discusses implications for therapy design, sequencing, and evaluation. Section 7 concludes by situating the framework as a complementary, geometry-first perspective for theoretical biology and medicine.

## 2. Counterfactual Manifolds

### 2.1. Path Space Formulation of Biological Dynamics

Consider a biological system interacting with an environment over a finite time horizon [*t*, *t + T*]. Let

(1) $$x_{s}\in X$$

denote the (generally unobserved) internal state of the system, and

(2) $$y_{s}\in Y$$

denote observable variables (e.g., neural signals, biomarkers, behavior).

We define the history up to time *t* as

(3) $$H_{t}≔\left\{y_{s}\;:s\leq t\right\}$$

We work on a trajectory (path) space

(4) $$T≔C\left(\left[t,\;t+T\right],\;\;\;\;\;\;X\times Y\right)$$

or, in discrete time with resolution ∆t,

(5) $$T_{∆t}≔{\left(X\times Y\right)}^{\left\{t,t+∆t,\ldots ,t+T\right\}}$$

Any stochastic dynamical system with internal memory induces a regular conditional probability measure

(6) $$q\left(\cdot |H_{t}\right)$$

on T, representing the law of future trajectories given the past [14].

Not an ensemble-of-trajectories framework. In standard stochastic-process, control-theoretic, or nonequilibrium formulations, the conditional path space law $q\left(\cdot |H_{t}\right)$ is treated as a distribution *generated by fixed effective dynamics*, and analysis focuses on properties of realized or sampled trajectories. The present framework differs in its explanatory target: the system’s adaptive dynamics act on the conditional future law itself, so the geometry of $q\left(\cdot |H_{t}\right)$—its diversity and sensitivity—is not merely an epiphenomenon of state evolution but a regulated object. Counterfactual instability, in our sense, therefore refers not to variability of trajectories under a fixed law, but to instability of the law of futures under self-modification. This distinction separates counterfactual geometry from attractors, invariant measures, and ensemble descriptions, which characterize generated behavior rather than the maintenance of access to unrealized alternatives.

No assumption is made that the system explicitly represents this distribution, that it is optimal, or that it is Bayesian. We only assume that the system’s effective dynamics admit such a conditional path space law—a minimal assumption satisfied by broad classes of stochastic processes in statistical physics, control, and nonequilibrium inference.

### 2.2. Definition of the Counterfactual Manifold

The fundamental object of interest is not a single realized trajectory, but the set of trajectories that remain probabilistically accessible to the system.

**Definition** **1 (Counterfactual Manifold).** *Let* $q\left(\cdot |H_{t}\right)$*be a probability measure on* T.

*A counterfactual manifold* $M_{t}\subset T$ *is any measurable set satisfying*

(7) $$q\left(M_{t}|H_{t}\right)\geq 1-\delta ,\;\;\;\;\;\;\;\;0<\delta \ll 1$$

*That is,* $M_{t}$ *is a credible/typical set of future trajectories.*

**Remark** **1 (No density threshold is assumed).** *The definition is invariant under reparameterization and does not rely on pointwise values of a density, which are ill-defined on continuous path spaces.*

**Remark** **2 (Non-uniqueness is intentional).** *As in large-deviation theory and statistical mechanics, many sets may satisfy the credibility condition [14,15]. The theory concerns coarse geometric properties shared across such sets, not their precise boundaries.*

**Remark** **3 (Finite-time object).** *No infinite-time or thermodynamic limit is assumed. When such limits exist, they may be used to define threshold-robust asymptotic quantities, but the framework does not depend on them.*

### 2.3. Statistical Manifold of Future Distributions

The geometry relevant for stability and sensitivity does not live directly on $M_{t}\subset T$, but on the family of distributions that generate it.

Assume that, locally in time, the conditional path measure belongs to a smoothly parameterized family

(8) $$\left\{q_{\theta }\left(\tau \right)\;:\theta \in \Theta \subset R^{d}\right\}$$

where θ represents internal degrees of freedom of the biological system (e.g., synaptic configurations, regulatory states, epigenetic variables).

For notational simplicity, the dependence on the conditioning history $H_{t}$ is absorbed into the parameter $\theta_{t}$; when considering the family $\left\{q_{\theta }\right\}$, the conditioning is held fixed.

Throughout the paper, we write $q_{\theta_{t}}$ as shorthand for $q\left(\cdot |H_{t}\right)$ when the conditioning history is summarized by internal parameters *θ*_t_.

The parameter space Θ forms a statistical manifold, equipped with the Fisher information metric

(9) $$g_{ij}\left(\theta \right)=E_{\tau \sim q_{\theta }}\left[\frac{\partial \log q_{\theta }(\tau )}{\partial \theta_{i}}\frac{\partial \log q_{\theta }(\tau )}{\partial \theta_{j}}\right]$$

provided standard regularity conditions hold (domination, differentiability, interchange of integration and differentiation) [16].

**Figure 2:**
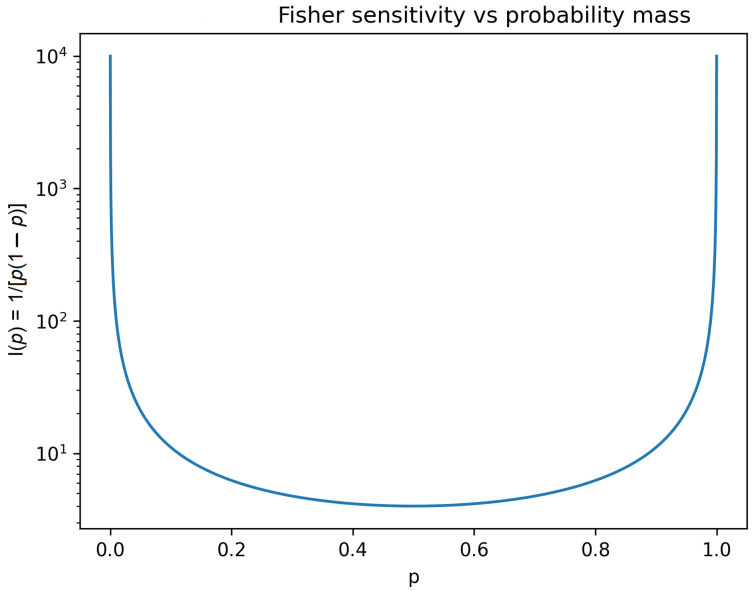
Fisher sensitivity vs. probability mass. For a minimal binary future distribution *q* = (*p*,1 − *p*), the Fisher information *I(p)* = 1/[*p*(1 − *p*)] diverges as *p* → 0 or *p* → 1. This divergence formalizes the geometric fragility associated with collapse of counterfactual structure: infinitesimal perturbations of internal parameters induce macroscopic changes in the distribution of future trajectories.

In the simplest nontrivial case of a binary future law *q* = (*p*,1 − *p*), the Fisher information reduces to *I(p)* = 1/[*p*(1 − *p*)], making explicit the divergence of sensitivity as probability mass concentrates near the boundary of the simplex (Figure 2). This illustrates how collapse of accessible futures is geometrically equivalent to fragility of future distributions [17].

This metric quantifies local distinguishability of future trajectory laws under infinitesimal perturbations of internal state parameters.

**Interpretation**

- High Fisher information:

Small changes in θ induce large changes in the distribution of futures → fragility.

- Low Fisher information:

Futures are insensitive to internal perturbations → robustness.

The geometry is thus a property of the space of future laws, not of individual trajectories.

### 2.4. Counterfactual Diversity via Relative Entropy

Shannon differential entropy on continuous path spaces is generally ill-defined or divergent. Instead, we define diversity relative to a reference process $q_{0}\left(\tau \right)$, representing a baseline or minimally structured dynamics (e.g., uncontrolled diffusion, prior physiology).

**Definition** **2 (Relative Counterfactual Entropy).** *We define the counterfactual diversity at time t as the path space Kullback–Leibler divergence*

(10) $$S_{t}≔D_{KL}\left(q\left(\tau |H_{t}\right)∥q_{0}(\tau )\right)$$

**Figure 3:**
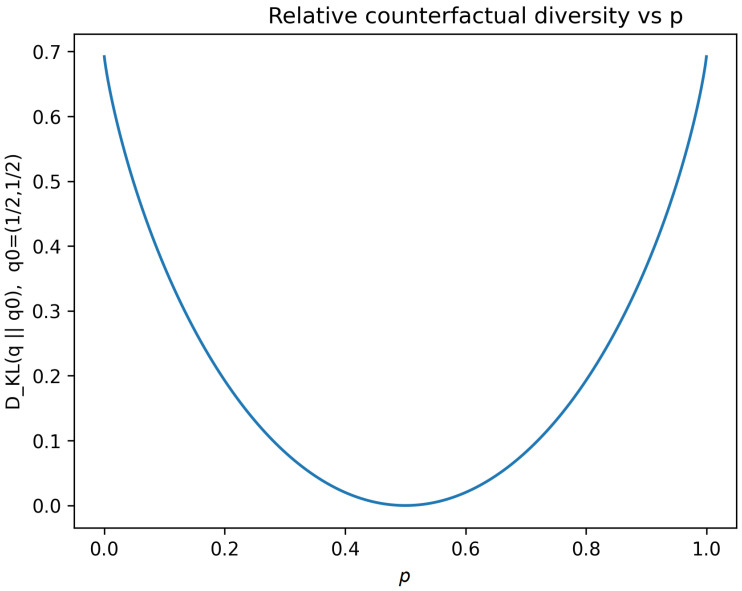
Relative counterfactual diversity vs. probability mass. Relative entropy *D_KL_*(*q*∥*q*_0_) for *q* = (*p*,1 − *p*) with baseline *q*_0_ = (1/2,1/2). Diversity is minimal at *p* = 1/2 and increases monotonically as probability mass concentrates toward a subset of futures, quantifying selective structure of the counterfactual manifold.

*For the same binary family, relative counterfactual diversity admits a simple closed form D_KL_(q* ∥*q*_0_*) with q*_0_ = (1/2,1/2), *which is minimized at maximal uncertainty and increases as futures become selectively restricted (Figure 3).*

This quantity is:

- coordinate-invariant,
- finite assuming $q\left(\cdot |H_{t}\right)$ is absolutely continuous with respect to $q_{0}$,
- standard in nonequilibrium statistical mechanics and stochastic control [18,19].

Interpretation:

- $S_{t}\approx 0$: futures indistinguishable from baseline → collapse of meaningful counterfactual structure.
- Large $S_{t}$: highly structured, selective future space.

Crucially, biological stability does not correspond to maximizing or minimizing $S_{t}$, but to maintaining it within a bounded, dynamically regulated range.

### 2.5. Temporal Evolution of the Manifold

Let $\theta_{t}$ denote the internal parameters at time *t*. We use $\theta_{t}$ as an effective internal coordinate on the space of conditional future laws. Then

(11) $$q\left(\cdot |H_{t}\right)=q_{\theta_{t}},\;\;\;{\dot{\theta }}_{t}=F\left(\theta_{t},H_{t}\right)$$

where *F* may be non-gradient, history-dependent, and stochastic.

As a result, the induced geometric quantities evolve in time:

(12) $$\frac{d}{dt}g_{ij}\left(\theta_{t}\right)\neq 0,\;\;\;\;\frac{d}{dt}S_{t}\neq 0$$

even in stationary external environments.

**Explanation of Equation (11).** The equality $q\left(\cdot |H_{t}\right)=q_{\theta_{t}}$ states that, at time *t*, the system’s conditional law over future trajectories is determined by its current internal parameters $\theta_{t}$, which summarize (possibly imperfectly) the information in the history $H_{t}$. In other words, $\theta_{t}$ is an *information state* (or sufficient summary in the approximate sense) that fixes the future path-law within a chosen family $\left\{q_{\theta }\right\}$. The second relation ${\dot{\theta }}_{t}=F\left(\theta_{t},H_{t}\right)$ expresses that these parameters evolve by an adaptive rule *F* that can depend on both the current parameter value and the accumulated history (e.g., via learning, plasticity, regulation). This is the key “self-modifying law” aspect: the system is not only generating trajectories under $q_{\theta_{t}}$, it is updating $\theta_{t}$, thereby changing the *future* distribution itself.

A discrete-time form makes the dependence explicit: $\theta_{t+∆t}=\theta_{t}+∆tF\left(\theta_{t},H_{t}\right)+\eta_{t}$ and $q\left(\tau_{t:t+T}|H_{t}\right)=q_{\theta_{t}}\left(\tau_{t:t+T}\right)$, where $\eta_{t}$ captures stochasticity in adaptation.

This places living systems in the class of nonequilibrium, self-modifying stochastic systems, where the law governing future trajectories is itself a dynamical object.

### 2.6. Observability and Coarse-Graining

Neither $M_{t}$ nor $q\left(\cdot |H_{t}\right)$ is directly observable. However, any observable map

(13) O:T→Z

induces a pushforward measure

(14) $$q_{O}≔q\left(\cdot |H_{t}\right)\circ O^{-1}$$

from which partial information about $\theta_{t}$, $g_{ij}$, and $S_{t}$ can be inferred.

This is directly analogous to inferring thermodynamic or transport properties from coarse observables without access to microscopic trajectories.

Minimal internal requirement (no explicit future model). The framework does not assume that a biological system explicitly represents $q\left(\cdot |H_{t}\right)$, the counterfactual manifold $M_{t}$, or any associated geometric quantities. The required assumption is weaker and purely dynamical: internal adaptive degrees of freedom must be sensitive to changes in the relative accessibility of alternative futures, so that shifts toward collapse (loss of accessible trajectories) or runaway sensitivity (excessive amplification of perturbations) feedback on the system’s own parameter updates. In this sense, the system need not encode futures as objects, but its regulation must depend on how its distribution of future responses changes under perturbation, as revealed through observable variability, sensitivity, or recovery statistics.

### 2.7. Minimal Assumptions and Falsifiability

The construction requires only:

1. stochastic dynamics;
2. a well-defined conditioning σ-algebra/information state (possibly summarized);
3. adaptive internal degrees of freedom.

No assumption of optimality, explicit inference, or goal-directed control is required.

The framework is falsifiable: If a biological system could be shown to maintain stable function while the induced conditional law over future trajectories exhibits a sustained collapse of counterfactual structure—operationalized, for example, by $S_{t}\to 0$ or an equivalent loss of probabilistically accessible futures—the counterfactual manifold hypothesis would fail.

### 2.8. Motivation for Adversarial Stabilization

The existence of a counterfactual manifold does not guarantee its stability. In the absence of regulatory structure, adaptive dynamics on Θ commonly lead, in broad classes of models, either to:

- degeneration (loss of counterfactual diversity), or
- divergence (unbounded sensitivity of future distributions).

In the next section, we show that adversarial coupling between expansion and constraint processes provides a generic mechanism for maintaining bounded geometry of future distributions.

Before introducing the stabilizing mechanism, it is important to clarify how the present framework differs from inference-based accounts such as the free energy principle, which are often invoked to explain anticipatory biological behavior.

**Relation to the Free Energy Principle.**

The free energy principle (FEP) has provided a powerful unifying framework for understanding biological systems as performing inference on hidden states of the world by minimizing variational free energy. Within this formulation, biological dynamics are organized around the reduction of present-state surprise or prediction error under a generative model of sensory data. Crucially, this limitation is structural rather than interpretive: no reparameterization of a scalar free energy objective can reproduce the saddle-point dynamics required for bounded, non-convergent regulation of future distributions.

The present framework is complementary in scope but fundamentally distinct in its primary object of description. Rather than focusing on inference over current or past states, it is concerned with the geometry and stability of distributions over future trajectories. This difference leads to non-overlapping notions of stability, variability, and pathology.

For clarity, these dimensions are listed explicitly.

Primary object. Under the free energy principle, the primary object of regulation is the surprise or variational free energy of present sensory states under a generative model. In counterfactual geometry, the primary object is the conditional distribution over future trajectories maintained by the system.

State space. The free energy principle operates on estimates of current latent states inferred from sensory data. By contrast, counterfactual geometry is defined on path space—that is, the space of unrealized but probabilistically accessible future trajectories.

Stability criterion. Within the free energy principle, stability corresponds to convergence toward minima of a scalar free energy functional. In counterfactual geometry, stability is defined as bounded, non-convergent dynamics on future distributions, allowing persistent regulation without fixation.

Role of variability. In inference-based formulations, variability is typically treated as noise to be explained, suppressed, or absorbed into uncertainty estimates. In counterfactual geometry, variability of future trajectories is itself a regulated quantity: excessive suppression leads to collapse of accessible futures, while excessive amplification produces instability.

Adaptation. Adaptation under the free energy principle consists in improved inference of the causes of sensory data through refinement of internal generative models. In the present framework, adaptation consists in stabilizing the space of accessible futures under continual perturbation and finite resource constraints.

Pathology. Within the free energy principle, dysfunction is associated with persistent inference failure or maladaptive priors. Within counterfactual geometry, pathology is identified with instability of counterfactual geometry itself, manifesting as collapse of future diversity, explosive sensitivity, or trapping within narrow subsets of trajectory space.

Importantly, this distinction is not a matter of interpretation but of formal expressivity. Because the FEP is formulated around optimization of a scalar functional of present-state beliefs, it does not natively represent properties of *future-distribution geometry*, such as bounded non-convergence, oscillatory stabilization, or sensitivity of unrealized trajectories. Conversely, the counterfactual framework introduced here does not replace inference-based accounts, but operates at a different descriptive level, addressing aspects of biological organization that are orthogonal to present-state optimality.

Importantly, this distinction does not imply incompatibility or contradiction. The present framework does not deny that biological systems may minimize variational free energy at the level of present-state inference. Rather, it identifies a distinct regulatory problem operating at a different descriptive layer: the stabilization of future-conditioned trajectory distributions under self-modification and finite resource constraints. While inference-based dynamics may operate within such systems, the maintenance of bounded, non-convergent counterfactual geometry cannot, in general, be reduced to optimization of a single scalar functional over present-state beliefs, because such functionals admit Lyapunov structure and therefore imply convergence or fixation. The adversarial stabilization described here is therefore orthogonal to inference-based descriptions and may coexist with them.

At the same time, free-energy-minimizing and gradient-flow formulations have proven highly successful in describing systems operating near equilibrium or in regimes characterized by convergence toward stable attractors. In such contexts, scalar-objective optimization provides both analytical tractability and strong empirical predictability. The present framework does not contest these successes. Rather, it addresses a distinct structural regime in which sustained bounded non-convergent regulation of future-conditioned distributions is functionally required.

This section establishes that (i) any adaptive biological system induces a conditional distribution over future trajectories; (ii) the relevant object for stability is the geometry of this distribution, not individual trajectories; and (iii) collapse or divergence of this geometry corresponds to loss of robustness or explosive sensitivity. These constructions rely only on minimal stochastic assumptions and are empirically falsifiable via observable proxies.

## 3. Adversarial Stabilization of Counterfactual Geometry

### 3.1. Instability of Unconstrained Adaptive Dynamics

We begin with a generic observation from dynamical systems and nonequilibrium statistical mechanics: adaptive systems that modify the law governing their own future trajectories are generically unstable [20].

Intuition (structural source of instability). When adaptive dynamics modify not merely a realized trajectory but the *future-generating law* itself, the update rule ${\dot{\theta }}_{t}=F\left(\theta_{t},H_{t}\right)$ feeds back on the mapping from internal parameters to future distributions. Small changes in *θ* can therefore reshape the distribution of accessible futures, which in turn alters subsequent adaptive signals derived from realized outcomes. This creates higher-order feedback: parameter updates influence the geometry of future possibilities, which then modifies future updates. In the absence of explicit stabilizing structure that penalizes boundary concentration or excessive sensitivity, such feedback generically drives the system either toward collapse (progressive restriction of accessible futures) or toward regions where the mapping *θ* ↦ *q*_θ_ becomes ill-conditioned, producing explosive sensitivity. Stability of counterfactual geometry is therefore not automatic under self-modification.

Let $\theta_{t}\in \Theta$ denote internal parameters governing the conditional path space distribution

(15) $$q_{\theta_{t}}\left(\tau \right)≔q\left(\tau |H_{t}\right)$$

Adaptive dynamics take the general form

(16) $${\dot{\theta }}_{t}=F\left(\theta_{t},H_{t}\right)$$

where *F* encodes learning, plasticity, regulation, or adaptation.

Absent explicit stabilizing structure, there is no general reason for such adaptive dynamics to preserve bounded diversity or bounded sensitivity of future distributions. In broad classes of adaptive systems, evolution of $q_{\theta_{t}}$ may lead to (i) concentration of probability mass on progressively narrower subsets of trajectory space (collapse of accessible futures), or (ii) growth of sensitivity of future distributions to parameter perturbations (explosive instability). We therefore treat stable counterfactual geometry as a non-generic property that requires explanation, rather than as a default outcome of adaptation.

### 3.2. Expansion–Constraint Decomposition

We assume that the adaptive flow admits a structural decomposition

(17) $${\dot{\theta }}_{t}=F^{\left(+\right)}\left(\theta_{t},H_{t}\right)+F^{\left(-\right)}\left(\theta_{t},H_{t}\right)$$

where

- $F^{\left(+\right)}$ promotes expansion of accessible futures (exploration, diversification);
- $F^{\left(-\right)}$ enforces constraint (energetic feasibility, structural consistency, historical limits).

This decomposition is functional rather than anatomical: the same biological substrate may implement both components at different times or scales.

To characterize their geometric effects, we use:

- relative counterfactual diversity

(18) $$S_{t}≔D_{KL}\left(q_{\theta_{t}}∥q_{0}\right)$$

- Fisher information magnitude

(19) $$ℶ\left(\theta_{t}\right)≔tr\;g\left(\theta_{t}\right)\;\;\;\;\;or\;\;\;\;\;\lambda_{max}\left(g\left(\theta_{t}\right)\right)$$

where $g\left(\theta \right)$ is the Fisher information matrix of the path-law family.

### 3.3. Limitations of Single-Objective Adaptive Flows

If adaptive dynamics are governed by a single scalar objective,

(20) $${\dot{\theta }}_{t}=-\nabla_{\theta }V\left(\theta_{t}\right)$$

then the resulting evolution is a gradient flow on parameter space. Under standard regularity and boundedness conditions, such flows admit *V* as a Lyapunov function and therefore cannot sustain recurrent or cyclic behavior. Trajectories typically approach the set of critical points of *V*, rather than exhibiting persistent nonequilibrium motion.

In the present context, convergence of $\theta_{t}$ corresponds to fixation of the conditional path space distribution $q_{\theta_{t}}$, implying loss of ongoing counterfactual variability and reduced capacity for adaptive response. Persistent diversity of accessible futures is therefore incompatible with purely single-objective gradient-based adaptation, except in the presence of externally imposed time dependence or noise.

**Proposition (informal).** Any adaptive dynamics governed by a single scalar objective over internal parameters admits a Lyapunov function and therefore cannot generically sustain bounded, non-convergent dynamics on the space of future trajectory distributions without external forcing or fine-tuned time dependence [21]. This implies that persistent regulation of counterfactual diversity and sensitivity is structurally incompatible with single-objective optimization.

### 3.4. Dual-Channel Adversarial Dynamics

To obtain intrinsically non-convergent yet bounded dynamics, we introduce a dual-channel antagonistic learning mechanism.

Let

- *θ* ∈ Θ parameterize expansion of counterfactual trajectories $q_{\theta }\left(\tau |H_{t}\right)$;
- *ϕ* ∈ Φ parameterize a feasibility functional *c_ϕ_(τ)* that scores trajectories according to energetic, structural, or historical constraints.

We define a saddle objective

(21) $$\underset{\theta }{\min}\;\underset{\phi }{\max}\;U\left(\theta ,\phi \right)$$

with representative form

(22) $$U\left(\theta ,\phi \right)=E_{\tau \sim q_{\theta }}\left[c_{\phi }(\tau )\right]-E_{\tau \sim q_{0}}\left[c_{\phi }\left(\tau \right)\right]+\lambda \Omega \left(\phi \right)-\beta R\left(\theta \right)$$

where $q_{0}$ is a reference path measure, Ω(*ϕ*) regularizes the constraint channel, and $R\left(\theta \right)$ promotes exploration.

The corresponding dynamics are

(23) $${\dot{\theta }}_{t}=-\nabla_{\theta }U\left(\theta_{t},\phi_{t}\right),\;\;\;\;\;\;\;{\dot{\phi }}_{t}=+\nabla_{\phi }U\left(\theta_{t},\phi_{t}\right)$$

which are not reducible to gradient flow of a single scalar potential. Such saddle dynamics are known to produce rotational components and persistent bounded motion even in simple model classes [22,23].

Clarification on the use of “adversarial”. The term *adversarial* is used here in a strictly formal and dynamical sense. It does not denote conflict, competition, or antagonism in a biological, evolutionary, or metaphorical sense, nor does it imply Darwinian selection between agents or subsystems. It also does not presuppose GAN-like architectures or any particular machine-learning implementation. Rather, *adversarial* refers to the presence of irreducible saddle-point structure in the adaptive dynamics governing future-conditioned distributions: opposing gradient directions acting on the *same object*—the distribution over possible futures—such that no single scalar objective exists whose minimization reproduces the coupled dynamics. This non-reducibility is essential: it is the mathematical source of bounded, non-convergent regulation of counterfactual geometry. Functional opposition may be implemented by overlapping or shared biological substrates and need not correspond to discrete modules, conflict, or competition. The term is therefore descriptive of the geometry of the learning dynamics, not of biological intent, strategy, or evolutionary struggle.

Saddle-point counterfactual regulation (design pattern). The coupled dynamics (23) instantiate a general design pattern, which we refer to as saddle-point counterfactual regulation: a dual-channel adaptive architecture in which expansion and constraint act on the *same future-conditioned distribution* through irreducibly non-gradient (rotational) dynamics. The defining feature of this pattern is not the specific form of *U(θ,ϕ)*, but the absence of any scalar Lyapunov function whose optimization reproduces the joint flow. This non-reducibility enables bounded, persistent regulation of future-distribution diversity and sensitivity without convergence to a fixed future law, and therefore provides a minimal sufficient mechanism for stabilizing counterfactual geometry under self-modification.

### 3.5. Resource-Gated Boundedness

Biological antagonistic adaptation operates under finite resources. To capture this constraint explicitly, we introduce a resource variable $r_{t}$ (e.g., metabolic capacity, synaptic gain, immune activation budget) coupled to the adversarial dynamics:

(24) $${\dot{r}}_{t}=\prod \left(r_{t};\theta_{t},\phi_{t},H_{t}\right)$$

with both the expansion objective and the constraint functional depending on $r_{t}$.

We assume that $r_{t}$ evolves on a bounded domain,

(25) $$r_{t}\in \left[0,r_{max}\right]$$

and that high expansion or constraint activity depletes available resources, inducing effective dissipation or saturation in the dynamics of $\left(\theta_{t},\phi_{t}\right)$. Under such conditions, excessive growth of sensitivity or diversity feeds back negatively through resource limitation, while excessive suppression relaxes as resource demand decreases.

Information–thermodynamic constraint (Landauer-type). The regulation of a conditional distribution over future trajectories requires maintaining and updating internal informational degrees of freedom $\theta_{t}$. Such updates generally involve logically irreversible operations (e.g., resetting, overwriting, or compressing internal states). According to Landauer’s principle, any logically irreversible operation performed by a physical system incurs a minimal energetic cost proportional to the amount of information erased.

While the present framework does not require a detailed thermodynamic model, this principle provides a physical grounding for the bounded resource variable $r_{t}$. Sustained regulation of counterfactual diversity and sensitivity cannot be energetically free: maintaining rich future repertoires and preventing runaway sensitivity both require continuous information processing under metabolic constraints.

In this sense, collapse-dominated regimes may be interpreted as energy-limited loss of accessible futures, while runaway sensitivity may reflect insufficient dissipative regulation of informational degrees of freedom. The bounded adversarial regime therefore corresponds to a dynamically sustainable balance between information maintenance and energetic constraint.

Finite resource availability therefore provides a natural mechanism by which the coupled adversarial dynamics can admit a compact attracting set in Θ_exp_ × Θ_con_, rendering bounded counterfactual geometry physically plausible without reliance on external forcing or fine-tuned parameter balance.

### 3.6. Geometric Failure Modes

Departures from adversarial stabilization manifest as distinct geometric signatures:

- Collapse: reduced effective support of $q_{\theta_{t}}$, often accompanied by decreases in $S_{t}$ or reduced effective rank of $g\left(\theta_{t}\right)$;
- Explosive sensitivity: growth of $ℶ\left(\theta_{t}\right)$, indicating heightened sensitivity of future distributions to perturbation;
- Mode trapping: persistence of global diversity with concentration on narrow subsets of $M_{t}$.

These are diagnostic signatures rather than strict equivalences and may vary across model classes.

### 3.7. A Minimal Adversarial Model of Counterfactual Instability and Stabilization

#### 3.7.1. Purpose of the Minimal Model

The role of the minimal model is not illustration but necessity: it demonstrates that adversarial optimization is sufficient for stabilization and that single-objective adaptation fails even in the simplest nontrivial setting.

Section 2 and Section 3 introduced counterfactual geometry and argued that stable biological function requires mechanisms capable of maintaining bounded diversity and bounded sensitivity of future distributions. We now demonstrate this claim constructively by introducing the minimal adversarial model that exhibits all three geometric regimes discussed above: collapse, explosive sensitivity, and bounded non-convergent regulation.

The purpose of this model is not biological realism, but mechanistic necessity: to show that (i) single-objective adaptation fails generically and (ii) explicit adversarial optimization over future distributions is sufficient to stabilize counterfactual geometry.

#### 3.7.2. Object of Control: Future Distributions

Let $T=\left\{\tau_{1},\tau_{2}\right\}$ denote a minimal set of possible future trajectories. The system’s counterfactual state is represented by a probability distribution

(26) $$q=\left(p,1-p\right)\in ∆\left(T\right)$$

where *q* parameterizes the relative accessibility of alternative futures.

In addition, we introduce a feasibility (constraint) function

(27) $$c=\left(c_{1},c_{2}\right)\in R^{2}$$

which assigns costs to trajectories. Importantly, *q* and *c* are controlled by distinct adaptive subsystems, reflecting the expansion–constraint decomposition introduced in Section 3.2.

#### 3.7.3. Explicit Adversarial Objective

We define a zero-sum game between two subsystems:

- an Explorer, which adapts *q* to preserve diversity of futures;
- a Verifier, which adapts *c* to enforce feasibility constraints.

The joint objective is

(28) $$\underset{q\in ∆\left(T\right)}{\min}\underset{c\in R^{2}}{\max}\;L\left(q,c\right)$$

with payoff

(29) $$L\left(q,c\right)=E_{r\sim q}\left[c\left(\tau \right)\right]-\beta H\left(q\right)-\lambda {\left\|c\right\|}^{2}$$

where

- $H\left(q\right)=-\sum_{i}q_{i}\log q_{i}$ is Shannon entropy (counterfactual diversity);
- β > 0 controls exploratory pressure;
- λ > 0 regularizes constraint magnitude.

This objective is irreducibly adversarial: the Explorer and Verifier optimize incompatible functionals of the *same future distribution*. There exists no scalar objective whose gradient descent reproduces both adaptations.

#### 3.7.4. Adversarial Learning Dynamics

We consider simultaneous gradient descent–ascent on L. To respect the simplex constraint on *q*, we use a mirror-descent (replicator) flow:

(30) $$\dot{p}=-p(1-p)\frac{\partial L}{\partial p}$$

while the constraint channel follows standard gradient ascent:

(31) $${\dot{c}}_{1}=\frac{\partial L}{\partial c_{1}},\;\;\;\;\;{\dot{c}}_{2}=\frac{\partial L}{\partial c_{2}}$$

Explicitly,

(32) $$\dot{p}=-p(1-p)\left[\left(c_{1}-c_{2}\right)-\beta \log\frac{p}{1-p}\right]\;{\dot{c}}_{1}=p-2\lambda c_{1}\;{\dot{c}}_{2}=\left(1-p\right)-2\lambda c_{2}$$

These equations define a genuine saddle-point flow on the space of future distributions and constraints.

**Figure 4:**
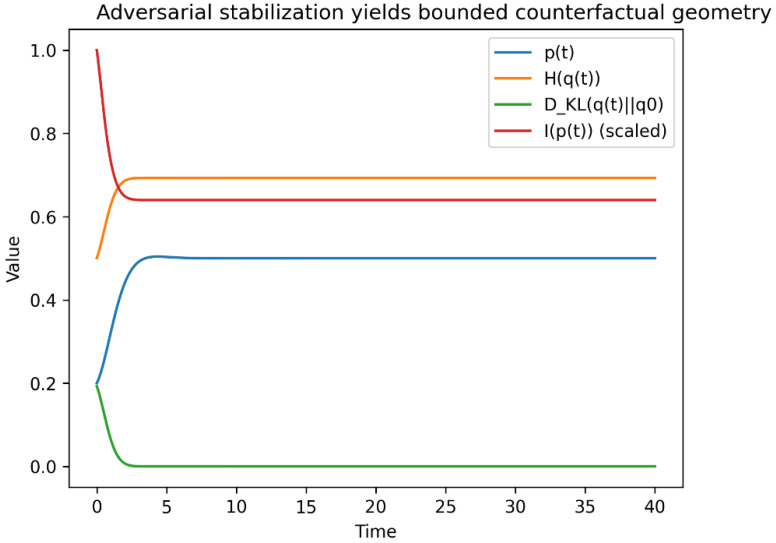
Adversarial stabilization yields bounded counterfactual geometry. Time evolution of the minimal adversarial model under intermediate (*β*, *λ*). Shown are the future probability *p*(*t*), counterfactual diversity *H(q(t))*, relative diversity *D_KL_*(*q(t)*∥*q*_0_) with *q*_0_ = (1/2, 1/2), and Fisher sensitivity *I*(*p*(*t*)) (scaled). After transient amplification, all quantities remain bounded, illustrating stabilization of future distributions without collapse or runaway sensitivity.

Figure 4 illustrates the resulting dynamics in the bounded adversarial regime. Despite transient sensitivity amplification, the future distribution remains interior to the simplex, with counterfactual diversity and Fisher sensitivity regulated to finite ranges. This demonstrates constructively that explicit adversarial coupling over future distributions is sufficient to stabilize counterfactual geometry.

#### 3.7.5. Geometry of the Dynamics

The system admits an interior stationary point

(33) $$p^{*}=\frac{1}{2},\;\;\;\;c_{1}^{*}=c_{2}^{*}=\frac{1}{4\lambda }$$

Linearization reveals that the Jacobian generically contains rotational components, reflecting the absence of a Lyapunov function. Consequently, the dynamics are not gradient-like and do not generically converge monotonically.

Depending on (*β, λ*), three regimes arise:

1. Collapse (*β* small):

*p(t)* → 0 or 1, entropy *H(q)* → 0, Fisher sensitivity diverges.

2. Explosive sensitivity (*λ* small):

constraint regulation weakens, perturbations in *c* induce large swings in *q*.

3. Bounded adversarial regulation (intermediate parameters):

*p*(*t*) remains in the interior, entropy and Fisher information remain bounded, and trajectories exhibit oscillatory or weakly damped motion.

These regimes correspond directly to the geometric failure modes defined in Section 3.6.

**Figure 5:**
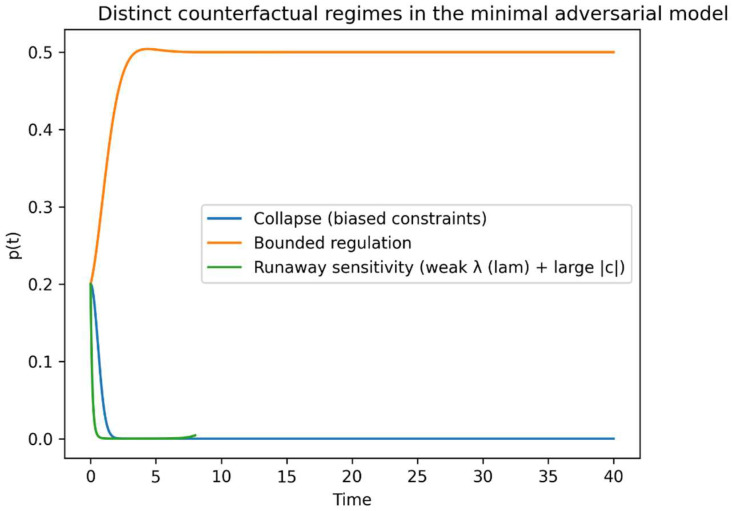
Distinct counterfactual regimes in the minimal adversarial model. Representative trajectories *p(t)* for three parameter regimes: collapse under persistent constraint bias, bounded adversarial regulation, and runaway sensitivity under weak constraint regularization with large constraint mismatch. These regimes correspond directly to the geometric failure modes defined in Section 3.6.

The three geometric regimes predicted by the analysis are explicitly realized by the minimal model under different parameterizations (Figure 5). Collapse corresponds to boundary attraction of the future distribution, bounded regulation to interior stabilization, and runaway sensitivity to rapid near-boundary excursions driven by weakened constraint regularization.

**Figure 6:**
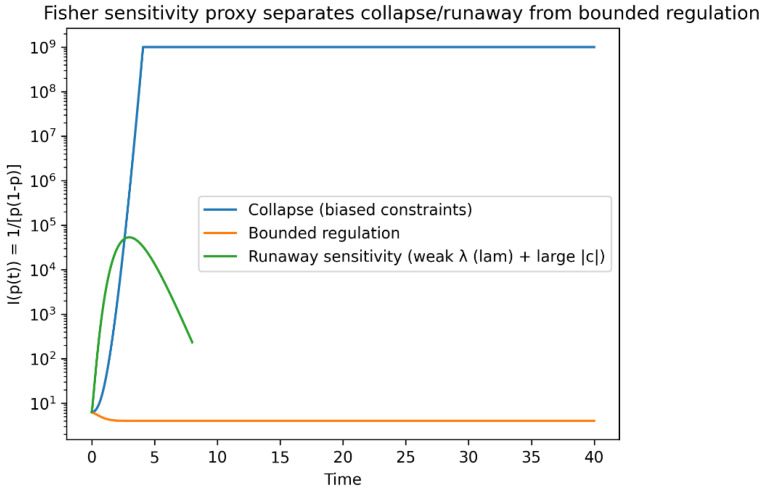
Fisher sensitivity proxy separates collapse and runaway from bounded regulation. Time evolution of Fisher sensitivity *I*(*p(t*)) = 1/[*p*(*t*)(1 − *p*(*t*))] on a logarithmic scale for the three regimes. Collapse and runaway sensitivity are characterized by extreme amplification of sensitivity due to boundary excursions, whereas bounded regulation maintains finite sensitivity throughout.

Although the three regimes may appear similar at the level of mean trajectories, they are sharply separated by sensitivity-based diagnostics. In particular, the Fisher information exhibits orders-of-magnitude divergence in collapse and runaway regimes, while remaining bounded under adversarial regulation (Figure 6).

#### 3.7.6. Interpretation in Terms of Counterfactual Geometry

In this minimal model

- counterfactual diversity is measured by *H*(*q*);
- relative diversity by *D*_KL_(*q*∥*q_0_*) with *q*_0_ = (1/2,1/2);
- future sensitivity by the Fisher information *I*(*p*) = 1/[*p*(*1* − *p*)].

Bounded adversarial dynamics correspond to regulation of all three quantities without convergence to a fixed future law. Collapse and explosion correspond to geometric pathologies of the counterfactual manifold.

#### 3.7.7. What the Model Establishes

This model establishes that

- stabilization of counterfactual geometry is not generic under single-objective adaptation;
- explicit adversarial optimization over future distributions is sufficient to produce bounded, non-convergent dynamics;
- the stabilizing mechanism arises from saddle-point structure, not from feedback alone.

No biological assumptions are required for these conclusions.

Intuition (why noise is insufficient). Injecting stochasticity into a single-objective adaptive flow does not, by itself, regulate counterfactual geometry. If damping or regularization dominates, noise produces fluctuations around an attractor, and the conditional future law effectively fixes, leading to progressive loss of accessible alternatives. If damping is weak, noise repeatedly drives the system toward boundary regions of parameter space where future distributions become highly sensitive to perturbation, amplifying instability. In neither case does noise introduce restoring structure that simultaneously bounds diversity and sensitivity at the level of the future distribution. Stabilization in the present sense requires dynamical opposition acting directly on the geometry of $q_{\theta_{t}}$, not merely stochastic perturbation of trajectories.

Role of stochasticity. It is important to emphasize that stochasticity alone does not substitute for adversarial structure. Adding noise to single-objective adaptive dynamics—such as stochastic exploration combined with damping or regularization—does not generically stabilize counterfactual geometry [24,25]. In such systems, noise either averages out under dissipation, leading to convergence and collapse of accessible futures, or amplifies sensitivity by repeatedly driving the system toward boundary regions of the future distribution, producing instability. In the absence of irreducible saddle-point structure, stochastic perturbations do not create sustained bounded regulation of diversity and sensitivity, but merely induce transient excursions around attractors or along unstable directions. The bounded, non-convergent regimes exhibited by the minimal model therefore arise from adversarial coupling over future distributions, not from stochasticity per se.

#### 3.7.8. Scope and Limitations

The model is intentionally minimal and does not claim biological realism or universality. Its role is to demonstrate that the geometric phenomena described in Section 2 and Section 3 arise from explicit adversarial learning on future distributions, and cannot be eliminated by reparameterization or reduction to a single scalar objective.

#### 3.7.9. Relation to Existing Dynamical Systems Frameworks

The counterfactual manifold introduced in this work is related to, but not reducible to, several established objects in dynamical systems theory. Clarifying these distinctions is essential for situating the present framework within existing mathematical and control-theoretic traditions.

First, attractor basins and invariant measures characterize asymptotic behavior of *realized* trajectories under fixed dynamics. By contrast, the counterfactual manifold concerns the *distribution of possible futures prior to their realization*, including trajectories that may never be visited. It is therefore defined over unrealized alternatives rather than over the empirical support of observed dynamics.

Second, viability kernels in control theory identify subsets of state space from which constraints can be satisfied indefinitely under admissible controls. The counterfactual manifold is not a subset of state space, but a probability geometry over future trajectories. Instability in the present framework arises not from direct violation of constraints, but from collapse or explosion of future diversity and sensitivity.

Third, ensembles of stochastic trajectories in nonequilibrium statistical physics describe distributions generated by fixed underlying dynamics. Here, by contrast, the geometry of the future distribution itself is the regulated object: adversarial dynamics act directly on the distribution of futures rather than merely generating it as an epiphenomenon of state evolution.

In this sense, the counterfactual manifold constitutes a distinct explanatory object: neither an attractor, nor a viability set, nor a stationary ensemble, but a geometric structure over unrealized futures whose stability is functionally relevant for adaptive behavior.

### 3.8. Operational Proxies and Falsifiable Predictions

This section specifies how counterfactual instability can be empirically detected and how the framework could be falsified using finite, noisy observations.

#### 3.8.1. Schematic Empirical Discrimination of Regimes

Although the present work is theoretical, the proposed operational proxies can already be illustrated using simulated data generated by the minimal adversarial model. Figure 5 and Figure 6 and Table 1 provide a concrete demonstration of how collapse, bounded regulation, and runaway sensitivity can be discriminated using finite observations of response diversity, sensitivity amplification, and boundary residence.

In the collapse regime, probability mass concentrates near the boundary of the future simplex, producing rapid loss of counterfactual diversity and extreme amplification of Fisher sensitivity. In the bounded regulation regime, the future distribution remains interior, with diversity and sensitivity confined to finite ranges. In the runaway sensitivity regime, transient near-boundary excursions generate large sensitivity spikes without permanent collapse. These distinctions are captured quantitatively by the diagnostics summarized in Table 1.

Importantly, regime identification does not require access to the full underlying state-space dynamics. The relevant quantities—diversity of responses across perturbations, sensitivity to small variations, and recovery geometry—are defined purely at the level of observable distributions. In biological settings, analogous measurements could correspond to variability of neural responses under repeated stimulation, diversity of immune repertoires following antigenic challenge, or recovery trajectories following therapeutic intervention.

This schematic example illustrates how the framework yields falsifiable predictions: systems occupying different regions of counterfactual geometry generate distinct, measurable signatures in response statistics. The same operational logic can therefore be applied to empirical data, even when mechanistic details are incomplete or unknown.

#### 3.8.2. Motivation

The counterfactual manifold and its geometric instabilities are defined on path space distributions that are not directly observable. To render the framework empirically testable, we require operational proxies: measurable quantities that reflect proximity to collapse, explosive sensitivity, or bounded adversarial regulation, using finite, noisy observations.

The goal is not exact reconstruction of counterfactual geometry, but diagnostic discrimination between regimes.

Table 1 summarizes numerical diagnostics of the three geometric regimes realized by the minimal model. These quantities—boundary residence, entropy loss, relative diversity, and sensitivity amplification—motivate the operational proxies introduced below and demonstrate how collapse, runaway sensitivity, and bounded regulation can be discriminated using finite observations.

#### 3.8.3. Perturbation–Response Sensitivity as a Proxy for Future Instability

Consider repeated applications of a controlled or naturally occurring perturbation *u(t)* to a biological system, and let *z(t)* denote an observable response (e.g., neural activity, biomarker level, behavioral output).

We define the effective counterfactual sensitivity

(34) $${ℶ}_{eff}≔\frac{Var\left(z|u\right)}{Var\left(u\right)}$$

where variances are estimated across repeated perturbation–response pairs under comparable conditions.

Interpretation:

- Low ${ℶ}_{eff}$: insensitivity to perturbation (collapse-dominated regime)
- High ${ℶ}_{eff}$: amplification of small perturbations (explosive sensitivity)
- Intermediate, bounded ${ℶ}_{eff}$: regulated counterfactual geometry

This quantity is a coarse observable surrogate for Fisher information–based sensitivity of future distributions.

#### 3.8.4. Response Diversity Under Matched Conditions

Let ${\left\{z_{i}\left(t\right)\right\}}_{i=1}^{N}$ denote responses to repeated presentations of nominally identical inputs or conditions. We define response diversity

(35) $$D_{resp}≔{Var}_{i}\left(F\left[z_{i}\left(t\right)\right]\right)$$

where F extracts a task- or domain-relevant summary (e.g., peak response, latency, trajectory embedding).

Interpretation:

- Low $D_{resp}$: restricted future repertoire (collapse)
- Excessively high $D_{resp}$: unregulated exploration or noise amplification
- Stable intermediate values: bounded counterfactual diversity

Importantly, $D_{resp}$ captures diversity of realized futures, not noise magnitude.

#### 3.8.5. Recovery Geometry Following Perturbation

Let the system be perturbed at time *t*_0_ and observed during recovery. Define a distance *d*(⋅,⋅) on observable state space and compute

(36) $$R\left(\tau \right)≔d\left(z\left(t_{0}+\tau \right),\bar{z}\right)$$

where $\bar{z}$ is a reference trajectory or baseline.

Characteristic signatures include:

- monotonic decay to baseline (collapse-dominated stabilization);
- divergence or prolonged excursions (explosive regimes);
- bounded oscillatory recovery (adversarially stabilized dynamics).

Recovery geometry provides a time-resolved signature of counterfactual stability without requiring explicit future sampling.

#### 3.8.6. Early-Warning Indicators

Because counterfactual instability concerns future distributions, changes in these proxies may precede shifts in mean values or structural damage. In particular:

- rising ${ℶ}_{eff}$ without increased diversity suggests approaching explosive sensitivity;
- declining *D*_resp_ with preserved mean response suggests impending collapse;
- increasing recovery time without loss of amplitude suggests weakening adversarial coupling.

These indicators provide candidate early-warning signals of disease progression or treatment-induced destabilization.

#### 3.8.7. Scope and Limitations

The proxies defined here are not unique and do not exhaust the space of possible measurements. They are intentionally low-dimensional, domain-agnostic, and compatible with existing experimental and clinical datasets. Their role is to enable regime identification, not full reconstruction of counterfactual manifolds.

#### 3.8.8. Continuous Finite-Sample Illustration via Resource-Selection Adversarial Learning

The schematic examples above establish that counterfactual regimes can be discriminated using finite-sample proxies in a minimal analytic setting. To demonstrate that these diagnostics are not artifacts of the discrete two-future construction, we provide a continuous finite-sample illustration based on explicit adversarial learning under resource selection.

In this toy environment, a generator *G_θ_* proposes future endpoints $x_{T}\in R$ from a latent noise source, while a discriminator *D_ϕ_* learns to distinguish these proposals from an environment-selected reference distribution defined by a resource-gated feasibility cost. The discriminator therefore represents adaptive environmental selection, while the generator adapts to survive selection under competing pressures of feasibility and diversity.

The resulting learning dynamics constitute an explicit saddle-point game over future distributions: the generator is rewarded for producing diverse futures that evade selection, while the discriminator sharpens feasibility constraints by preferentially admitting low-cost trajectories. No single scalar objective governs the joint dynamics.

Across parameter regimes controlling feasibility pressure, diversity pressure, and resource limitation, the system robustly exhibits three qualitative behaviors corresponding to the geometric regimes identified in Section 3.6 and Section 3.7: (i) collapse, in which generated futures concentrate narrowly within the feasible region; (ii) runaway sensitivity, in which diversity pressure overwhelms constraint, driving probability mass toward or beyond feasibility boundaries; and (iii) bounded regulation, in which future distributions remain broad yet feasible without converging to a single endpoint.

**Figure 7:**
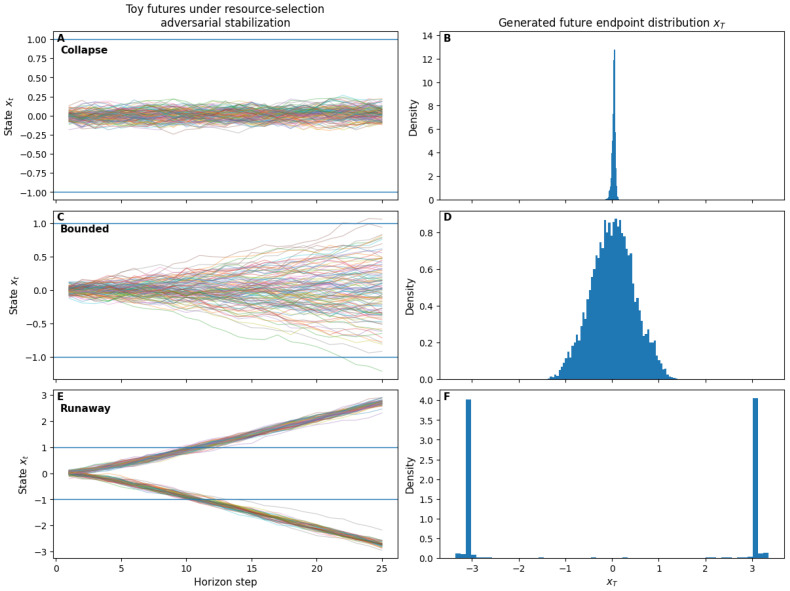
Empirical signatures of counterfactual instability and adversarial stabilization. Representative realizations of the minimal adversarial model illustrating three geometric regimes of future dynamics. In the collapse regime (**A**,**B**), future trajectories rapidly concentrate, producing loss of diversity and low sensitivity to perturbation. In the bounded regulation regime (**C**,**D**), adversarial coupling between expansion and constraint maintains a diverse yet feasible set of futures with bounded sensitivity and rapid recovery. In the runaway sensitivity regime (**E**,**F**), weakened constraint allows excessive exploration, leading to large sensitivity amplification, frequent boundary violations, and delayed recovery. Shown are sample future trajectories (**left**) and the corresponding distributions of future endpoints (**right**), from which the finite-sample diagnostics reported in Table 2 are computed.

Figure 7 shows representative endpoint distributions and sampled toy futures for these regimes. Table 2 reports finite-sample diagnostics computed from generated samples, including response diversity, entropy proxy, perturbation sensitivity amplification, boundary residence, and feasibility cost. These quantities cleanly separate the three regimes using only observable statistics, without access to latent dynamics or analytic expressions for Fisher information.

Importantly, this illustration does not introduce additional theoretical assumptions. It serves only to verify that the operational proxies defined in Section 3.8.3, Section 3.8.4, Section 3.8.5 and Section 3.8.6 discriminate counterfactual instability regimes in a continuous, noisy, finite-sample setting governed by explicit adversarial dynamics. The same logic therefore applies directly to empirical systems in which only sampled responses to perturbation are available.

Implementation details and full hyperparameter specifications are provided in Appendix A.

#### 3.8.9. Candidate Biological Datasets and Empirical Extraction of Proxies

Although the present framework is theoretical, many existing biological experimental paradigms already generate the repeated-perturbation structure required to estimate the proposed operational proxies. The key requirement is not access to latent internal states, but repeated observations of system responses under controlled or naturally varying perturbations.

Neural systems. In electrophysiology, MEG/EEG, calcium imaging, or fMRI experiments, repeated presentations of identical or slightly jittered stimuli provide trial-to-trial response ensembles. From such data:

- Response diversity can be estimated as dispersion of neural population trajectories in a low-dimensional embedding (e.g., PCA or manifold projection).
- Perturbation sensitivity can be quantified by amplification of small stimulus variations into variability of neural responses.
- Recovery geometry can be computed as time-resolved distance from baseline activity following stimulus offset.

Collapse-dominated regimes would manifest as progressively stereotyped responses with reduced trajectory dispersion; runaway sensitivity would manifest as disproportionate amplification of small stimulus perturbations and prolonged recovery excursions; bounded regulation would show intermediate diversity with finite sensitivity and structured return dynamics.

Immune systems. Longitudinal B-cell or T-cell receptor sequencing across vaccination, infection, or immune challenge provides direct access to repertoire diversity and concentration. Similarly, ex vivo stimulation assays (e.g., cytokine panels under graded perturbation) yield repeated response distributions. Here:

- diversity proxies correspond to clonal richness and dispersion across challenges;
- sensitivity proxies correspond to amplification of graded stimulation into cytokine output variance;
- boundary residence may correspond to sustained concentration of repertoire into narrow clonal subsets or chronic constraint-dominated states.

Collapse regimes correspond to repertoire narrowing or exhaustion; runaway regimes to hypersensitive inflammatory amplification; bounded regimes to sustained diversity with finite responsiveness.

Oncology and ecological systems. Time-series measurements of tumor burden, phenotypic heterogeneity, or ecological population abundances under cyclic therapy or environmental shifts similarly permit proxy extraction. Repeated perturbations (e.g., therapy cycles, environmental shocks) generate response ensembles. Collapse appears as loss of phenotypic heterogeneity and monotonic stabilization; runaway sensitivity appears as extreme variance amplification or frequent boundary excursions (e.g., rapid resistance emergence); bounded regulation appears as controlled oscillatory or adaptive response under perturbation.

Importantly, regime discrimination requires only finite-sample statistics of perturbation–response variability and recovery dynamics. Full reconstruction of underlying state-space dynamics or explicit estimation of Fisher information on path space is not required. The proposed proxies are therefore compatible with existing datasets in neuroscience, immunology, oncology, and ecology, and can be computed retrospectively from repeated-measurement designs already common in these fields.

### 3.9. Scope of the Mechanism

The analysis above does not establish adversarial stabilization as a universal or necessary property of all adaptive systems. Instead, it identifies dual-channel antagonistic learning as a sufficient and parsimonious design pattern by which adaptive, nonequilibrium systems can sustain bounded counterfactual diversity and sensitivity under finite capacity and resource constraints.

Alternative stabilizing mechanisms may exist in restricted settings or under externally imposed regulation. The present framework therefore emphasizes recurrence and explanatory power, rather than theorem-level universality.

### 3.10. Transition to Biological Interpretation

In biological systems, the expansion–constraint decomposition may be realized through competition between neural populations, opposing regulatory pathways, ecological feedback loops, or energetic and structural limits. The specific implementation is system-dependent and need not correspond to a fixed anatomical partition.

What is shared across domains is a functional requirement: adaptive systems that internally generate futures under finite resources require stabilizing feedback that counteracts unchecked expansion or collapse of accessible trajectories. In the following section, we examine how distinct biological domains instantiate this structure, and how characteristic disease states may be interpreted as failures of adversarial stabilization acting on counterfactual geometry.

## 4. Disease as Counterfactual Instability

### 4.1. From Component Failure to Geometric Pathology

Traditional medical models characterize disease in terms of dysfunctional components, aberrant molecular pathways, or deviations of measurable variables from normative ranges. While such descriptions are indispensable for mechanistic and therapeutic detail, they provide limited insight into *why* distinct diseases exhibit shared features such as rigidity, volatility, loss of adaptability, or unpredictable response to intervention.

Within the present framework, disease is not defined by the failure of specific components, but by instability of counterfactual geometry: the inability of an adaptive biological system to maintain bounded diversity and bounded sensitivity of its future trajectory distribution under finite resources. This perspective reframes disease as a property of dynamical organization, rather than of structure alone.

### 4.2. Counterfactual Instability as a Unifying Principle

Let $q_{\theta_{t}}\left(\tau \right)$ denote the conditional path space law governing future trajectories, and let $S_{t}$ and $ℶ\left(\theta_{t}\right)$ denote relative counterfactual diversity and Fisher information magnitude, respectively (Section 2 and Section 3).

We define counterfactual instability as the failure to keep these quantities within a bounded, dynamically regulated regime. Importantly, instability does not require monotonic divergence; it may arise through fixation, oscillatory trapping, or intermittent excursions.

From this viewpoint, diverse disease processes correspond to a small number of geometric failure modes, independent of organ system or molecular substrate [26].

### 4.3. Primary Geometric Failure Modes

#### 4.3.1. Collapse of Counterfactual Diversity

In collapse-dominated regimes, adaptive dynamics drive the future distribution toward a restricted subset of trajectories. Formally, this corresponds to a reduction in the effective support of $q_{\theta_{t}}$, often accompanied by a decrease in $S_{t}$ or loss of effective dimensionality of the statistical manifold.

Functionally, collapse manifests as:

- reduced behavioral or physiological repertoire;
- diminished responsiveness to perturbation;
- progressive rigidity despite ongoing environmental change.

Such regimes are consistent with degenerative processes, chronic inflammatory exhaustion, and states of pathological fixation, though the framework does not assert a one-to-one mapping between geometric mode and clinical diagnosis.

#### 4.3.2. Explosive Sensitivity

In contrast, explosive regimes are characterized by growth of sensitivity of future distributions to small perturbations, reflected in increases of $ℶ\left(\theta_{t}\right)$. Here, infinitesimal changes in internal parameters or external inputs produce disproportionately large shifts in accessible futures.

Functionally, this regime is associated with:

- instability of response;
- unpredictability of outcomes;
- amplification of noise or minor perturbations into macroscopic effects.

Such behavior is observed in acute dysregulation, uncontrolled proliferation, and hypersensitive physiological states. Importantly, explosive sensitivity does not require high diversity; a narrow but unstable future distribution can also produce pathological volatility.

#### 4.3.3. Mode Trapping and Fragmentation

A third class of failure arises when counterfactual diversity persists globally, but probability mass becomes trapped in narrow regions of trajectory space. In this regime, $S_{t}$ may remain moderate, yet realized trajectories repeatedly explore the same limited subset of futures.

This manifests as:

- repetitive or stereotyped dynamics;
- cycling without genuine exploration;
- resistance to interventions that do not alter the underlying geometry.

Mode trapping highlights that disease cannot be inferred from diversity alone; the structure of the manifold matters, not just its volume.

### 4.4. Disease as Breakdown of Adversarial Stabilization

Section 3 identified dual-channel antagonistic learning as a mechanism capable of stabilizing counterfactual geometry. Within that framework, disease arises when this stabilization fails in one of three broad ways:

1. Expansion dominance

Constraint mechanisms fail to regulate exploration, leading to excessive sensitivity or runaway adaptation.

2. Constraint dominance

Expansion mechanisms are suppressed or resource-starved, leading to collapse of accessible futures.

3. Coupling failure

Expansion and constraint subsystems remain active but become decoupled or misaligned, producing fragmentation or trapping.

Crucially, these failures are *dynamical*, not structural: the same anatomical substrate may transition between regimes over time.

### 4.5. State Dependence of Intervention Effects

A central implication of this framework is that the effect of an intervention cannot be characterized independently of the system’s counterfactual geometry at the time of application.

Formally, an intervention acts as:

- a perturbation of the path-law parameters *θ*_t_,
- a modification of constraint parameters *ϕ*_t_,
- or an impulse on the resource variable *r*_t_.

The same intervention may therefore:

- restore bounded dynamics in one regime,
- exacerbate instability in another,
- or produce qualitatively distinct outcomes depending on timing and state.

This provides a principled explanation for context-dependent efficacy, paradoxical treatment responses, and delayed or nonlinear therapeutic effects observed across medicine.

### 4.6. Beyond Diagnostic Categories

Because counterfactual instability is defined at the level of future distributions rather than symptoms or organs, it naturally cuts across traditional diagnostic boundaries. Disorders conventionally classified as unrelated may share geometric failure modes, while clinically similar syndromes may arise from distinct instabilities.

This does not invalidate existing classifications, but reframes them as phenomenological projections of deeper dynamical regimes. In this sense, counterfactual geometry offers a substrate on which diverse mechanistic models can be compared and integrated.

### 4.7. Testable Implications

Interpreting disease as counterfactual instability yields several testable predictions:

- Pathological states should be associated with measurable changes in variability, sensitivity, or response diversity, rather than solely mean values.
- Effective interventions should shift geometric diagnostics toward bounded regimes, even if traditional biomarkers transiently worsen.
- Early disease progression may be detectable as changes in counterfactual geometry before overt structural damage appears.

These predictions are agnostic to specific molecular mechanisms and can, in principle, be evaluated across biological scales.

By reframing disease as instability of counterfactual geometry, this framework shifts the focus of medicine from correcting components to stabilizing futures. In the following section, we examine how this perspective can be concretely instantiated in specific biological domains, and how domain-specific interventions act as geometric operators on the space of possible trajectories.

## 5. Domain Instantiations of Counterfactual Stabilization

### 5.1. General Principle

The preceding sections introduced counterfactual geometry and adversarial stabilization as abstract dynamical constructs. Here we illustrate how these constructs can be instantiated across biological domains—not by positing shared mechanisms, but by showing that diverse systems can be described within a common geometric and dynamical language.

Across domains, three functional elements recur: (i) processes that generate alternative future trajectories, (ii) processes that evaluate or constrain those trajectories, and (iii) finite resource or capacity limits that bound their interaction. While the biological substrates differ, these functional roles are conserved.

### 5.2. Neural Systems: Cognition and Psychiatric Disease

In neural systems, counterfactual generation arises from internally generated activity not directly driven by sensory input, including spontaneous cortical dynamics, predictive simulation, imagination, and planning. Constraint is imposed through inhibitory control, error signaling, neuromodulatory regulation, and energetic limits.

From this perspective, cognition depends not on accurate prediction of a single future, but on maintaining a structured ensemble of plausible futures whose diversity and sensitivity remain bounded. Neural variability, metastability, and transient synchronization are natural signatures of such bounded counterfactual dynamics.

Within this framework, psychiatric disorders are interpreted as failures of counterfactual regulation rather than disorders of content. Collapse-dominated regimes correspond to restricted future repertoires, manifesting as rigidity, anhedonia, or perseveration; explosive sensitivity produces hypersensitivity to perturbation, volatility, and loss of coherence; and mode trapping corresponds to persistent cycling through narrow cognitive or affective states despite preserved global variability. This interpretation complements descriptive psychiatry by explaining why symptom clusters cut across diagnoses and why treatment effects are strongly state-dependent.

### 5.3. Immune Systems: Defense, Tolerance, and Autoimmunity

The adaptive immune system explicitly generates vast repertoires of potential responses through stochastic recombination and mutation, constituting a counterfactual space of possible immune futures, most of which are never realized. Constraint is imposed through selection, tolerance checkpoints, energetic costs, and regulatory signaling, which limit not only which responses are activated but which remain available.

Immune pathologies can be interpreted as failures of counterfactual stabilization. Collapse of repertoire diversity leads to immunodeficiency or exhaustion; excessive expansion or sensitivity produces autoimmunity or hyperinflammatory states; and fragmentation or misalignment of constraint mechanisms results in inappropriate targeting or chronic activation. This framing naturally unifies defense and tolerance as complementary aspects of the same geometric regulation problem.

### 5.4. Cancer: Evolutionary Exploration Under Constraint

Cancer progression involves continual exploration of phenotypic and genotypic space under strong selective pressure. Tumors maintain distributions over possible future states—such as drug resistance, invasion strategies, and metabolic adaptations—shaped by microenvironmental constraints.

From this perspective, cancer is not merely uncontrolled growth, but runaway counterfactual expansion under corrupted or weakened constraint channels [27]. Therapeutic interventions alter counterfactual geometry by removing regions of trajectory space (cell death), modifying constraints (e.g., microenvironmental change), or reshaping resource availability. Failure arises when therapy inadvertently increases future sensitivity or fragments the counterfactual manifold, thereby promoting resistance.

### 5.5. Development and Aging

Development can be interpreted as controlled narrowing of counterfactual space. Early in development, systems exhibit high diversity and sensitivity; over time, accumulating constraints stabilize specific trajectories while preserving limited adaptability. Premature collapse impairs function, whereas insufficient constraint leads to instability.

Aging is associated with reduced capacity to maintain bounded counterfactual geometry, manifesting as shrinking future repertoires, increased fragility to perturbation, and impaired recovery from shocks. Rather than a single mechanism, aging reflects degradation of the processes that previously stabilized counterfactual dynamics across systems.

### 5.6. Shared Predictions and Limits

Despite mechanistic differences, the framework yields cross-domain predictions: pathology should correlate more strongly with changes in variability, sensitivity, and response diversity than with mean values alone; early dysfunction may be detectable as geometric changes prior to overt structural damage; and effective interventions should restore bounded counterfactual dynamics even if short-term markers worsen. These predictions motivate shared measurement strategies across fields traditionally studied in isolation.

Domain instantiations are interpretive mappings, not proofs. The framework does not claim that all diseases reduce to the same mechanism or that all biological systems implement counterfactual stabilization identically. Instead, it provides a shared dynamical language in which domain-specific models can be compared, integrated, or distinguished.

#### Worked Example: Neural Circuit Under Repeated Sensory Perturbation

Experimental situation. Consider a cortical circuit in an animal or human subject repeatedly presented with the *same weak sensory stimulus* (e.g., a brief auditory tone or visual flash), under otherwise stable conditions. Population neural activity *z*(*t*) is recorded using electrophysiology or calcium imaging across many trials.

Observable measurements. Across repeated trials, three quantities are extracted:

1. Response diversity: the variability of population activity trajectories across trials (e.g., variance or dispersion in a low-dimensional neural embedding).
2. Perturbation sensitivity: the amplification of small variations in stimulus timing or amplitude into variability of neural responses.
3. Recovery geometry: how neural activity returns to baseline following stimulus offset (e.g., monotonic decay versus oscillatory or metastable relaxation).

Observed pattern. Suppose that mean firing rates and stimulus-evoked response amplitudes remain unchanged, but:

- trial-to-trial response diversity steadily decreases,
- responses become increasingly stereotyped, and
- recovery trajectories become fast and monotonic with little fluctuation.

Geometric interpretation. Within the present framework, this pattern indicates collapse-dominated counterfactual geometry: the conditional distribution over possible future neural trajectories has contracted onto a narrow subset of responses. The system appears stable in terms of average behavior, but has lost access to alternative future dynamics.

Intervention and reassessment. A neuromodulatory intervention (e.g., increasing cortical gain or excitation) is applied. Following intervention, response diversity increases, but small stimulus variations now produce disproportionately large changes in neural activity, and recovery becomes prolonged and erratic.

Revised geometric interpretation. Despite increased variability, the system has shifted into an explosive sensitivity regime, not a healthy one: the counterfactual manifold has expanded without sufficient constraint, leading to instability rather than restored adaptability.

Stabilizing outcome. By contrast, an intervention that restores balance between excitatory and inhibitory regulation increases response diversity *while keeping sensitivity bounded* and recovery oscillatory or metastable. This pattern corresponds to bounded, adversarially stabilized counterfactual geometry, interpreted here as restoration of adaptive function.

Key point. At no stage is it necessary to reconstruct latent neural states, identify attractors, or predict specific future trajectories. The inference concerns the *regime of counterfactual geometry* inferred from observable perturbation–response statistics, and interventions are evaluated by how they move the system between regimes rather than by their effects on mean responses alone.

Having established counterfactual instability as a coherent lens across biological domains, we now turn to intervention. In the next section, we examine how medical treatments—pharmacological, surgical, radiative, and behavioral—can be understood as geometric operators acting on the space of biological futures, and how this perspective reshapes the logic of therapy design and evaluation.

## 6. Interventions as Geometric Operators on Biological Futures

### 6.1. From Causal Correction to Geometric Action

Medical interventions are conventionally described in causal terms—drugs inhibit pathways, surgery removes tissue, radiation damages DNA, stimulation excites or suppresses activity. While mechanistically indispensable, such descriptions do not explain why identical interventions yield qualitatively different outcomes across patients, disease stages, or treatment sequences.

Within this framework, interventions are reinterpreted as operators acting on counterfactual geometry. Rather than asking how a component is altered, we ask how the space of accessible future trajectories is reshaped—by modifying diversity, sensitivity, or the coupling between expansion and constraint mechanisms. This shift embeds causal reasoning within a higher-level dynamical description rather than replacing it.

### 6.2. Classification of Intervention Operators

Let $q_{\theta_{t}}\left(\tau \right)$ denote the conditional path space distribution over future trajectories. An intervention applied at time *t_0_* induces a transformation

(37) $$q_{\theta_{t_{0}^{-}}}\to q_{\theta_{t_{0}^{+}}}$$

which can be classified into three operator types.

Projection operators remove regions of trajectory space entirely (e.g., surgical resection, cytotoxic therapies, ablative radiation). Geometrically, they project the counterfactual manifold onto a lower-dimensional subset, eliminating futures that pass through the removed region. Such operators can stabilize expansion-dominated regimes but risk inducing collapse or excessive rigidity when diversity is already limited.

Constraint-modifying operators alter feasibility without directly removing trajectories (e.g., metabolic modulation, immune checkpoint manipulation, neuromodulatory drugs). These reshape the feasibility functional *c_ϕ_*(*τ*), modifying which futures are penalized or permitted. Their effect depends critically on pre-intervention geometry: tightening constraints may stabilize explosive regimes or exacerbate collapse-dominated ones.

Resource-redistribution operators act on the resource variable *r_t_* governing expansion–constraint dynamics (e.g., vascular normalization, metabolic support or deprivation, modulation of synaptic gain or arousal). These operators do not directly change trajectories or constraints, but alter regulatory capacity, often producing delayed or nonlinear effects.

### 6.3. Timing and State Dependence

Because interventions act on geometry rather than static components, their effects are inherently state-dependent.

Here, “state” refers to the system’s current position in the space of conditional future laws, that is, its present counterfactual geometry (e.g., whether diversity is collapsed, bounded, or sensitivity is amplified). An intervention is therefore an operator mapping one future distribution to another. Because stability is defined geometrically rather than component-wise, the same intervention may increase boundedness in one region of this space (e.g., runaway sensitivity) while worsening instability in another (e.g., collapse). The effect depends on the pre-intervention geometry, not only on the mechanistic direction of the intervention. Formally, an intervention acts as *q_θ_*_−_ → *q_θ_*_+_, altering diversity and sensitivity metrics rather than merely modifying a component variable.

The same operator applied at different points on the statistical manifold may restore bounded dynamics, induce instability, or shift the system into a different failure regime. This provides a principled explanation for paradoxical drug responses, delayed toxicity, resistance following initial success, and irreversibility caused by premature intervention. Timing is therefore a dynamical variable, not merely a practical consideration.

### 6.4. Combination and Sequencing

Sequential interventions compose as operators on counterfactual geometry:

(38) $$q\overset{O_{1}}{\to }\;q^{\prime }\overset{O_{2}}{\to }q^{\prime \prime }$$

Operator composition is generally non-commutative: $O_{2}\circ O_{1}\neq O_{1}\circ O_{2}$ [28]. This explains why treatment order matters, why induction therapies alter responses to maintenance therapies, and why washout periods can reverse or amplify effects. Optimal treatment design therefore cannot be reduced to independent optimization of individual components.

### 6.5. Radiotherapy as a Geometric Sculptor

Radiotherapy provides a clear example of geometric intervention. Localized radiation simultaneously removes families of trajectories (cell death), alters feasibility via vascular and immune effects, and redistributes resources by modifying tissue microenvironment. Its impact depends on spatial gradients, timing, and coupling to biological constraints, explaining pseudoprogression, abscopal effects, state-dependent side effects, and sensitivity to fractionation and sequencing—phenomena not captured by dose-based metrics alone.

### 6.6. Therapeutic Success and Failure Redefined

Within this framework, therapeutic success is defined not by elimination of pathology, normalization of biomarkers, or short-term response, but by restoration of bounded counterfactual dynamics. Failure arises when interventions over-collapse future diversity, amplify sensitivity, decouple expansion and constraint mechanisms, or irreversibly fragment the manifold. Short-term improvement may therefore coincide with long-term destabilization, explaining the frequent failure of surrogate endpoints to predict durable outcomes.

### 6.7. Implications for Clinical Trial Design

Interpreting interventions as geometric operators implies that trials should measure variability, sensitivity, and recovery dynamics in addition to mean responses; stratify patients by pre-intervention geometric state rather than diagnosis alone; and employ adaptive, sequential designs to probe operator composition. This reframing does not invalidate existing methodologies but exposes their limitations [28].

The geometric operator framework does not claim that all therapeutic effects reduce to a small set of geometric parameters, nor that mechanistic models are obsolete. Rather, it provides a unifying dynamical layer in which mechanistic effects acquire context and directionality.

By reframing interventions as operators acting on the geometry of biological futures, this perspective shifts therapy from correcting components to stabilizing adaptive dynamics. The concluding section summarizes the implications of this shift and outlines how a geometry-first perspective reshapes the conceptual foundations of medicine.

## 7. Conclusions

Modern medicine has achieved extraordinary mechanistic detail, yet continues to struggle with variability, unpredictability, and context dependence of disease and treatment. These limitations are not solely empirical; they reflect a deeper conceptual gap. By treating biological systems primarily as collections of components or pathways, prevailing frameworks often overlook the fact that living systems are defined not only by their present state, but also by the structure of their possible futures.

In this work, we introduced a geometric formulation of biological function grounded in the structure and stability of future-conditioned trajectory distributions. By formalizing the concept of a counterfactual manifold and its stabilization through dual-channel antagonistic learning, we reframed health as the maintenance of bounded diversity and bounded sensitivity of biological futures, and disease as characteristic instabilities of this geometry. This perspective is not intended to replace mechanistic explanation, but to situate it within a higher-order dynamical organization that governs adaptability, robustness, and failure across scales.

A geometry-first view offers a unifying perspective on several persistent challenges in medicine. It helps explain why identical interventions may yield divergent outcomes, why timing and sequencing can dominate dosage, and why short-term improvement can coexist with long-term destabilization. It also clarifies why diseases traditionally treated as distinct may share deep dynamical structure, while clinically similar syndromes may arise from fundamentally different instabilities. In this framework, interventions act not merely as causal corrections, but as operators that reshape the space of accessible futures, with effects that depend irreducibly on the pre-intervention geometry of the system.

The implications are not merely philosophical; they are operational. A geometry-first perspective motivates new observables beyond static biomarkers, trial designs sensitive to dynamical state, and therapeutic goals centered on restoring adaptive stability rather than enforcing nominal normality. It also provides a principled foundation for integrating diverse modalities—pharmacological, surgical, radiative, behavioral—within a single dynamical language.

Scope of applicability. The framework developed here is not intended as a universal characterization of all adaptive or biological systems. Its claims apply specifically to systems that satisfy three structural conditions: (i) the system internally generates a conditional distribution over multiple possible future trajectories rather than realizing a single fixed path; (ii) this future-conditioned distribution is itself modified by the system’s internal dynamics over time; and (iii) the generation and regulation of futures operate under finite resource or capacity constraints. Only systems meeting these conditions face the stabilization problem analyzed in this work and therefore admit adversarial—or formally equivalent non-gradient—dynamics as a sufficient mechanism for maintaining bounded counterfactual geometry. Systems that lack any of these properties—such as purely reactive systems, systems with fixed future laws, or systems regulated entirely by externally imposed constraints—fall outside the scope of the theory.

This work does not claim universality, nor does it propose a single mechanism for all disease. Instead, it offers a minimal, testable framework for understanding why living systems so often fail in similar ways despite radically different substrates. By shifting attention from components to futures, and from equilibrium to stability of motion, a geometry-first medicine reframes one possible way of thinking about diagnosis, intervention, and recovery.

The task ahead is not to abandon existing models, but to embed them within this broader geometric perspective where appropriate. If successful, such an integration could support a transition in medicine analogous to earlier shifts in physics—from forces to fields, from trajectories to phase space—providing a conceptual foundation on which both theory and practice may continue to evolve.

The contribution of this work is therefore not a universal mechanism, but a formal constraint: any system that maintains adaptive function under uncertainty must regulate the geometry of its future distributions, by adversarial or equivalent non-gradient means.

## Figures and Tables

**Table 1:** **Regime diagnostics linking observables to counterfactual failure modes.** Summary statistics for collapse, bounded regulation, and runaway sensitivity regimes, including boundary attraction, entropy loss, relative diversity, Fisher sensitivity amplification, and fraction of time spent near the boundary of the future simplex. These diagnostics motivate the operational proxies defined in Section 3.8 and provide concrete signatures of counterfactual instability.

Regime	Min *p*	Max *p*	Min *H*(*q*)	Max *H*(*q*)	Max KL(*q*||*q*_0_)	Max *I*(*p*)	Boundary Time Fraction
Collapse (biased constraints)	≈0	0.20	≈0	0.50	0.69	10^9^	0.98
Bounded regulation	0.20	0.50	0.50	0.69	0.19	6.25	0.00
Runaway sensitivity (weak λ + large |c|)	≈0	0.20	≈0	0.50	0.69	5 × 10^4^	0.98

**Table 2:** Finite-sample regime diagnostics for adversarial counterfactual dynamics. Summary statistics computed from generated future endpoint samples under three parameter regimes of the resource-selection adversarial model. Collapse is characterized by vanishing diversity, low sensitivity, and concentration well within feasibility bounds. Bounded regulation maintains intermediate diversity and sensitivity with minimal boundary residence. Runaway sensitivity exhibits extreme variance, sensitivity amplification, persistent boundary violation, and delayed recovery. All quantities are estimated from finite samples and require no access to latent dynamics or analytic future distributions.

Regime	Diversity Proxy Var(x_t_)	Entropy Proxy H(x_t_)	Sensitivity Amplification (RMS/δz)	Boundary Residence	Mean Feasibility Cost	Recovery Time Proxy
Collapse	0.0008	3.34	0.05	0.000	0.000	0
Bounded	0.1785	3.72	0.48	0.011	0.000	0
Runaway	9.3902	1.24	13.15	0.992	18.75	25

## Data Availability

The original contributions presented in this study are included in the article. Further inquiries can be directed to the corresponding author.

## Abbreviations

The following abbreviations are used in this manuscript:

**Symbol**	**Meaning**
*t*,*T*	Current time and finite future horizon
*x_s_*∈*X*	Internal (latent) system state at time *s*
*y_s_*∈*Y*	Observable variables at time *s*
*ℋ_t_*	Observation history up to time *t*
τ	Future trajectory
δ	Path (trajectory) space over [*t*, *t* + *T*]
*𝒯*	Conditional law over future trajectories
*q* (·|*ℋ_t_)*	Parameterized family of future trajectory distributions
*q_θ_*	Internal parameters governing future law
*θ_t_* ∈ Θ	Counterfactual manifold (typical set of futures)
*𝓜_t_*	Credibility tolerance for $M_{t}$
*g*_ij_(*θ*)	Fisher information metric on Θ
ℶ(*p*)	Fisher information for binary future distribution
B(*θ*)	Scalar Fisher sensitivity summary
*q*_0_	Reference (baseline) path space measure
*S_t_*	Relative counterfactual diversity *D_KL_*(*q*∥*q*_0_)
*H(q)*	Shannon entropy (finite discrete cases only)
*F*, *F*^(+)^, *F*^(−)^	Adaptive flow; expansion and constraint components
*β*	Exploratory (diversity-promoting) parameter
*λ*	Constraint regularization parameter
*c*, *c*_i_	Feasibility/constraint scores
*r*_t_	Resource or capacity variable
*ℬ**_eff_*	Empirical perturbation–response sensitivity
*D*_resp_	Response diversity proxy
*R*(τ)	Recovery geometry metric
FEP	Free energy principle

## Appendix A. Continuous Adversarial Illustration: Model, Algorithm, and Parameters

This appendix provides the full specification of the continuous finite-sample adversarial illustration referenced in Section 3.8.8. The goal of this illustration is not biological realism or empirical validation, but constructive sufficiency: to demonstrate that the operational proxies defined in Section 3.8 discriminate counterfactual instability regimes in a continuous, noisy setting governed by explicit adversarial (saddle-point) dynamics.

### Appendix A.1. Model Components

The toy system consists of two adaptive components acting on distributions over future endpoints $x_{T}\in R$:

1. Generator G_θ_

A parameterized stochastic map $x_{T}=G_{\theta }(z)$, where z ~ N(0,I), inducing a future distribution $q_{\theta }(x_{T})$.

2. Discriminator D_ϕ_

A scalar-valued function trained to distinguish generator samples from samples drawn from an environment-selected reference distribution.

The discriminator represents adaptive environmental selection rather than a competing agent. The generator adapts to survive selection while balancing feasibility, diversity, and resource pressure.

### Appendix A.2. Environment-Selected Reference Distribution

Environmental feasibility is defined via a soft cost

$$c\left(x\right)={\left[\max\left(\left|x-c_{0}\right|-\omega ,0\right)\right]}^{p}+\epsilon (x),$$

where

- c_0_ is the feasible region center;
- ω its half-width;
- *p* ≥ 2 controls boundary sharpness;
- ϵ(x) is a weak interior cost ensuring non-uniformity inside the feasible region.

An environment-selected reference distribution is defined implicitly as

$$p_{env}(x)\propto \exp\left(-\alpha c(x)\right)$$

with selection sharpness α > 0. Samples are obtained via importance resampling from a broad proposal distribution.

### Appendix A.3. Adversarial Learning Dynamics

The discriminator is trained to classify:

- environment-selected samples as real,
- generator samples as fake.

The generator minimizes a composite objective:

$$L_{G}=L_{adv}+\lambda E\left[c\left(x_{T}\right)\right]+\gamma E\left[{\left(x_{T}-c_{0}\right)}^{2}\right]-\beta D\left(q_{\theta }\right)$$

where

- $L_{adv}$ encourages survival under selection;
- λ controls feasibility pressure;
- γ controls resource limitation/frugality;
- β promotes diversity;
- D is a diversity proxy (variance or pairwise distance).

Crucially, no scalar Lyapunov function governs the joint dynamics: generator and discriminator follow opposing gradients on the space of future distributions, yielding irreducible saddle-point structure.

### Appendix A.4. Algorithm (Pseudocode)

This algorithm defines a genuine adversarial game over future distributions, not a single-objective optimization with noise.

**Algorithm A1** Resource-Selection Adversarial Stabilization (Toy Model)
Initialize generator parameters θ Initialize discriminator parameters φ for iteration = 1 to N do # --- Environment selection --- Sample candidate points x_prop ~ broad proposal Compute feasibility cost c(x_prop) Resample x_env ∝ exp(-α c(x_prop)) # --- Discriminator update --- Sample z ~ N(0, I) x_gen ← Gθ(z) Update φ to maximize: log Dφ(x_env) + log(1 − Dφ(x_gen)) # --- Generator update --- Sample z ~ N(0, I) x_gen ← Gθ(z) Compute: feasibility penalty c(x_gen) diversity proxy D(qθ) resource cost (x_gen − c0)^2 Update θ to minimize: adversarial loss +λ · feasibility penalty +γ · resource cost −β · diversity end for

### Appendix A.5. Hyperparameters and Regimes

All simulations reported in Section 3.8.8 and Table 2 use the following fixed settings unless otherwise stated.

**Table A1:** Simulation parameters.

Parameter	Meaning	Value
Latent dimension	dim(z)	8
Batch size	—	512
Training steps	—	8000
Critic steps per iteration	—	3
Learning rate (G, D)	—	2 × 10^−4^
Feasible center	c_0_	0
Feasible half-width	w	1
Boundary power	P	4

**Table A2:** Regime-specific parameters.

Regime	α (Selection)	λ (Feasibility)	β (Diversity)	γ (Resource)
Collapse	30.0	15.0	0.02	5.0
Bounded	30.0	15.0	0.35	0.5
Runaway	30.0	0.2	0.80	0.0

These parameter choices are not tuned for performance, but selected to cleanly separate geometric regimes.

### Appendix A.6. Diagnostics Computed

From generated samples, the following finite-sample diagnostics are computed:

- Response diversity: Var(x_T_)
- Entropy proxy: histogram-based Shannon entropy
- Sensitivity amplification: finite-difference RMS response to latent perturbation
- Boundary residence: fraction of samples outside feasibility band
- Recovery time proxy: return to feasible dominance after transient destabilization

All diagnostics depend only on sampled outputs and are directly measurable in empirical systems.

### Appendix A.7. Scope and Interpretation

This continuous illustration introduces no new theoretical assumptions beyond those already stated in Section 2 and Section 3. It serves solely to demonstrate that:

1. adversarial stabilization is realizable in continuous spaces;
2. counterfactual regimes persist under noise and finite sampling;
3. the proposed proxies discriminate regimes without access to latent dynamics.

The model is therefore a verification device, not a biological model.
